# Inner Speech during Silent Reading Reflects the Reader's Regional Accent

**DOI:** 10.1371/journal.pone.0025782

**Published:** 2011-10-19

**Authors:** Ruth Filik, Emma Barber

**Affiliations:** School of Psychology, University of Nottingham, Nottingham, United Kingdom; University of Groningen, The Netherlands

## Abstract

While reading silently, we often have the subjective experience of inner speech. However, there is currently little evidence regarding whether this inner voice resembles our own voice while we are speaking out loud. To investigate this issue, we compared reading behaviour of Northern and Southern English participants who have differing pronunciations for words like ‘glass’, in which the vowel duration is short in a Northern accent and long in a Southern accent. Participants' eye movements were monitored while they silently read limericks in which the end words of the first two lines (e.g., glass/class) would be pronounced differently by Northern and Southern participants. The final word of the limerick (e.g., mass/sparse) then either did or did not rhyme, depending on the reader's accent. Results showed disruption to eye movement behaviour when the final word did not rhyme, determined by the reader's accent, suggesting that inner speech resembles our own voice.

## Introduction

While reading silently, we often have the subjective experience of inner speech, or a “voice inside our heads”. However, there is currently little empirical evidence for this phenomenon, in particular, concerning the question of whether the voice in our heads that we experience during silent reading resembles our own voice while we are speaking out loud. The aim of the current study is to exploit prosodic differences in regional accents in developing a novel approach to investigate this issue. Specifically, we compare reading behaviour of Northern and Southern English participants who have differing pronunciations for words like ‘glass’, in which the vowel duration is typically short in a Northern accent (e.g., rhyming with ‘mass’) and long in a Southern accent (e.g., rhyming with ‘sparse’).

Previous research investigating the recognition of words in isolation suggests that phonological information is activated when participants are reading silently [1]–[8]. In addition, a number of eye movement studies have examined the activation of phonological information during sentence reading [9]–[16]. A prominent view in the literature asserts that the phonological information activated during reading does not necessarily involve detailed phonetic information (or the “sounding out” of words in a manner similar to external speech), but instead relies on more abstract, or impoverished, phonological codes (see e.g., [17], for discussion). However, results from some studies [18], [19] suggest that the phonological representations activated during reading may instead resemble external speech. Indeed, a number of studies have demonstrated that various prosodic factors, such as metrical structure and prosodic phrasing, can influence word-level processing, parsing, and interpretive processes during silent reading [20]–[26].

However, to our knowledge, it remains an open question to what extent the experience of inner speech resembles the external voice of the individual reader. We report an experiment that will examine whether the “voice in the head” resembles the reader's own voice by creating the anticipation for a word with a particular pronunciation, as determined by the reader's regional accent. Specifically, we will record eye movement behavior whilst participants with different regional accents (silently) read limericks such as (1) and (2) below:

1. There was a young runner from Bath, Who stumbled and fell on the path; She didn't get picked, As the coach was quite strict, So he gave the position to Kath.
2. There was an old lady from Bath, Who waved to her son down the path; He opened the gates, And bumped into his mates, Who were Gerry, and Simon, and Garth.

Limericks are poetic devices in which the final word (e.g., Kath/Garth) is expected to rhyme with the end words of lines 1 and 2 (e.g., Bath, path). In the current study, the materials differ in terms of whether the final word would rhyme, depending on the participant's regional accent. For participants with a short vowel pronunciation of words such as Bath/path, which is typical of speakers from the North of England, the final word rhymes for limerick 1 (Kath) but not for limerick 2 (Garth). In contrast, for participants with a long vowel pronunciation, which is typical of speakers from the South of England, the final word rhymes for limerick 2 but not limerick 1.

Thus, it is possible to create materials in which the final word of the limerick would either match, or mismatch, with the reader's expectation for a rhyme (see also [23]), depending on their regional accent. If regional accents are reflected in inner speech, as well as in external speech, we would expect to observe these mismatch effects in terms of disruption to the eye movement record during silent reading when participants encounter the final word of the limerick. On the other hand, if inner speech does not share the same phonetic properties as external speech, that is, does not mirror the individual reader's regional accent, we would expect to observe no such mismatch effects. Results showed more disruption to the eye movement record during reading for mismatch than match conditions, suggesting that regional accents are reflected in inner speech.

## Methods

### Ethics statement

This research was approved by the School of Psychology Ethics Committee at the University of Nottingham, and was conducted in accordance with British Psychological Society ethical guidelines. All participants gave written informed consent prior to taking part.

### Participants

Twenty-six native British-English speaking volunteers (13 with short vowel pronunciation and 13 with long vowel pronunciation) from the University of Nottingham community participated in the study (15 females, 11 males, mean age = 20.2).

### Materials and design

Twenty-four limericks were constructed, 12 in which the final word was designed to rhyme for short vowel participants only (e.g., 1, above) and 12 of which were designed to rhyme for long vowel participants only (e.g., 2). Thus, the stimulus materials either matched, or mismatched with the participant's expectation for a rhyme, as determined by their regional accent.

The stimulus file also contained 36 filler limericks. Six of these limericks had the final word altered so that it no longer rhymed, but not in a way that would be affected by the participant's accent, in order to draw attention away from the specific experimental manipulation.

Stimuli were presented double spaced in Courier 14 point font (bold), as black text on a white background, on a computer monitor 56 cm from participants' eyes. Three characters subtended approximately 1° of visual angle. Screen resolution was set to 1026×768, and the initial character of the limerick was positioned 250 pixels horizontally and 260 pixels vertically from the top left-hand corner of the screen.

### Procedure

Eye movements were recorded via an SR Research Eyelink 1000 eye-tracker, which sampled eye position every millisecond. Viewing was binocular, but only the right eye was recorded. Before the start of the experiment, the procedure was explained and participants were instructed to read normally and for comprehension. Participants were seated at the eye-tracker and placed their head on a chin and forehead rest to minimize head movements. Participants then completed a calibration procedure. Before the start of each trial, a fixation box appeared in the upper left quadrant of the screen. Once the participant fixated this box the stimulus computer displayed the target text. If the participant's apparent point of fixation did not match with the fixation box then the experimenter re-calibrated the eye-tracker. When the participant had finished reading each item, they looked away from the text to a post-it note that was affixed to the right-hand edge of the monitor, and then pressed a button. This was to ensure that reading times for the final word were always terminated by a saccade, rather than a button press. A comprehension question was displayed following one third of trials. A correct response rate of 94% indicated that participants were engaged in the task. Following the eye-tracking study, participants were asked what their home town was, and were then asked to read a list of words (path, grass, etc.) into a microphone in order to ensure that the stimulus words were indeed pronounced with consistently short or long vowels.

## Results

### Regions

**Figure 1:**
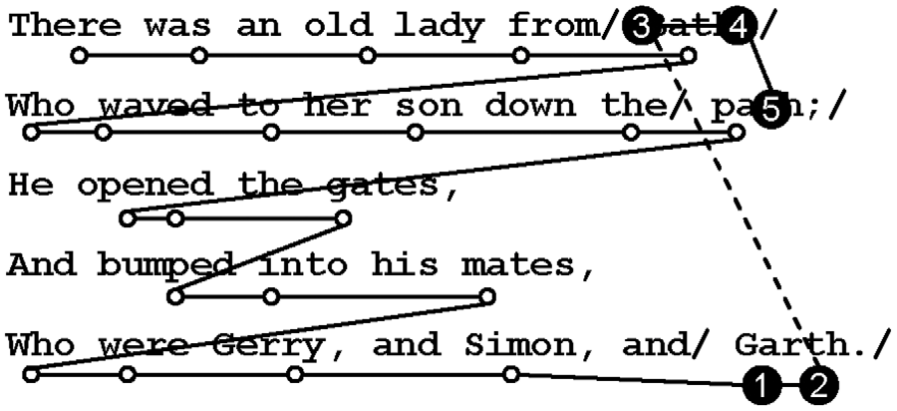
Sample eye movement trace illustrating the disruption experienced at the end of a trial (in this case for a short vowel participant reading a limerick designed to rhyme for long vowel participants only). Analysis regions are denoted by forward slashes. Circles represent fixations, and lines represent saccadic eye movements. Circles 1 and 2 represent the gaze duration on the critical word. The dashed line represents a first-pass regression out of the critical word, and a regression in to the end word of line 1. Circles 3 and 4 represent second-pass reading times for the end word of line 1, and circle 5 represents second-pass reading times for the end word of line 2. Circles 1, 2, 3, 4, and 5 represent regression path (or go-past) reading times for the critical word.

Materials were divided into regions for analysis (see Figure 1). Measures of eye movement behavior are reported for the *critical* region, which comprised the final word of the limerick (e.g., Kath, Garth). The critical word would either rhyme or not rhyme with the end words in the first two lines depending on the participant's accent. Participants' tendencies to go back and re-inspect these earlier words (e.g., Bath, path) were also examined.

### Analysis

An automatic procedure pooled short contiguous fixations. Fixations under 80 ms were incorporated into larger adjacent fixations within one character. Fixations of less than 40 ms and not within three characters of another fixation were deleted, as were fixations over 800 ms [27].

Five measures of reading behavior are reported (see Figure 1 for illustrations). In order to assess whether readers experienced difficulty on encountering the critical word in mismatching conditions, we examined *gaze duration*, *first-pass regressions out*, and *regression path* reading times for this word. *Gaze duration* is the sum of all the fixations made in a region until the point of fixation exits the region either to the left or to the right (also known as *first-pass reading time* when the region comprises more than a single word). *First-pass regressions out* indicates the proportion of trials where readers looked back from the region to an earlier piece of text between the time when the region was first entered from the left to the time when the region was first exited to the right. *Regression path* (or *go-past*) reading time is the sum of fixations from the time that a region is first entered until a saccade transgresses the right region boundary (or until the participant has finished reading, if the region of interest is the final region). This measure includes fixations made to re-inspect earlier portions of text and is usually taken to reflect early processing difficulty along with (at least some) time spent re-inspecting the text in order to recover from such difficulty. To further examine readers' behaviour in terms of re-inspecting the initial lines of the limerick, as well as re-inspecting the critical word, we report *second-pass reading times* for the end words of the first and second lines and for the critical word, and *regressions in* for the end words of the first and second lines. *Second-pass reading time* sums the duration of the fixations in a region after having left it either to the left or the right. *Regressions in* reflects the proportion of trials in which a reader made a regressive eye movement into the region, and provides an indication of the probability of re-inspecting a particular portion of text. In cases where the region had values of zero in gaze duration and regression path reading times, the relevant point was excluded from the analysis, and means were calculated from the remaining data points in the design cell. This procedure resulted in data losses of 15%.

Data for each region were subjected to two paired-samples t-tests (match vs. mismatch), treating participants (*t*_1_) and items (*t*_2_) as random variables (see Table 1 for descriptive statistics). Cohen's d is reported as a measure of effect size.

**Table 1:** Eye movement measures for regions of analysis (reading times are in ms, regressions are in %).

	Match		Mismatch	
	M	SE	M	SE
*Critical Region*				
Gaze duration	326	17.5	340	22.9
Regression path reading time	674	59.9	856	84.7
First-pass regressions out	41.8	4.4	48.3	4.0
Second-pass reading time	48.6	12.3	83.7	19.2
*End word of line1*				
Regressions in	5.2	1.5	10.3	2.7
Second-pass reading time	59.9	14.5	68.0	13.7
*End word of line 2*				
Regressions in	5.8	1.7	11.9	2.9
Second-pass reading time	40.9	9.6	80.0	19.7

### Critical Region

There were no significant effects in gaze duration (ts<1.4). However, regression path reading times were significantly longer in the mismatch condition than in the match condition, t1(25) = 3.82, d = .43, p<.005; t2(23) = 4.58, d = .81, p<.001. In support of this, the same pattern was evident in first-pass regressions out, t1(25) = 2.02, d = .30, p = .05; t2(23) = 1.22, d = .33, p = .24, significant by participants but not by items, and in second-pass reading times, t1(25) = 1.85, d = .42, p = .08; t2(23) = 2.72, d = .62, p<.05, significant by items and approaching significance by participants.

### End word of line 1

There were significantly more regressions in to the end word of the first line of the limerick in the mismatch than in the match condition, t1(25) = 2.80, d = .46, p<.05; t2(23) = 2.61, d = .68, p<.05. However second-pass reading times revealed no significant effects (ts<1).

### End word of line 2

There were significantly more regressions in, t1(25) = 3.04, d = .49, p<.01; t2(23) = 2.57, d = .72, p<.05, and significantly longer second-pass reading times, t1(25) = 2.45, d = .48, p<.05; t2(23) = 3.33, d = .79, p<.01, for the end word of the second line of the limerick in mismatch than match conditions.

## Discussion

In sum, the results indicate more disruption to the eye movement record when participants read limericks in which the final word did not match with their anticipated pronunciation, based on their own regional accent. This effect was evident in longer regression path reading times, more first-pass regressions out, and longer second-pass reading times for the final word of the limerick, as well as more regressions in to, and more time spent re-inspecting earlier portions of the limerick with which the final word would be expected to rhyme (i.e., the end words of lines 1 or 2).

Although the role of phonology in reading has been extensively examined, relatively few studies have investigated the exact nature of the implied speech representations, specifically, whether they are similar to overt speech, or instead are more abstract. The rationale underlying the current study was that if inner speech during silent reading resembles overt speech, then it should exhibit similar phonetic variations; in particular, it should share features of the reader's regional accent when they are speaking out loud. The mismatch effect present in the current data would support this notion.

Other evidence that inner speech resembles overt speech comes from “visual tongue-twister” effects, which show that tongue-twister sentences (e.g., “a bucket of blue bug's blood”) are difficult to read and prone to errors even when reading is silent, suggesting that word beginnings, at least, are clearly “articulated” in inner speech [19], [28], [29]. However, some of the evidence from tongue twister studies [30] seems to suggest that inner speech is impoverished at the featural level, compared to overt speech (but see [31]). The current data suggest that vowel length information is also represented (see also [9], [12]). Most importantly, however, the current study suggests that inner speech is influenced by the nature of the external speech of the individual reader, specifically, their regional accent.

In addition some previous studies have presented evidence to suggest that ‘person-particular’ knowledge of the author of a piece of text can influence reading of that piece of text. For example, it has been demonstrated that knowledge of the presumed author's speaking speed can influence how quickly people read aloud a passage of text [32]. This finding has also been replicated, and extended to silent reading [33]. Findings from other studies examining auditory imagery during reading have suggested that readers simulate aspects of the voices of the characters featured in the text (see [34], and also [35], for related findings). The current research supports, and extends these findings, by demonstrating that in the absence of information about the writer's voice, or that of characters involved in the text, inner speech during silent reading resembles the reader's own voice.

In relation to the wider literature on auditory imagery, recent work has focused on the common neural substrates underlying both auditory perception and imagery using music-related stimuli [36], [37], [38], language-related stimuli [39], [40], and environmental sounds [41]. Results have highlighted overlapping neural mechanisms underlying perception and auditory imagery in both the secondary and primary auditory cortex [42], [43]. The current work may also be of relevance to Brain Computer Interface researchers who investigate EEG markers of inner speech [44] with the goal of using inner speech to control external devices [45], and for researchers investigating inner speech in relation to auditory hallucinations in disorders such as schizophrenia [46], [47].

Although the data reported here support the notion that inner speech during silent reading resembles overt speech, there are a few points that need to be considered. In the current paper, limericks were adopted as experimental materials in order to create conditions leading to a match or mismatch in the anticipated pronunciation of the final word, based on the reader's regional accent. It could be argued that inner speech may be more likely to occur whilst reading limericks than during other reading tasks. However, this use of limericks does not detract from the conclusion that inner speech, when activated, reflects aspects of the reader's regional accent. Nevertheless, current research in our lab aims to investigate the influence of the reader's regional accent during different reading tasks. In addition, we aim to study the extent to which readers simulate the accents of characters mentioned in the text.

It is also possible that inner speech during reading limericks is of a qualitatively different nature than that experienced during other reading tasks. However, it is difficult to imagine a reason why readers would only adopt a regional accent whilst reading limericks, specifically. In support of this assumption, previous research suggests that mismatch effects relating to the prosodic stress patterns activated during silent reading of limericks is similar to that adopted whilst reading other text [23]. It should also be noted that the participant groups studied were relatively small (although not unusually small for studies of eye movements during reading). Finally, the current research was not designed to assess whether inner speech is involved in the word recognition process, and thus cannot speak to the debate regarding whether inner speech is necessary for language comprehension.

In conclusion, the current findings suggest that the inner speech experienced during silent reading reflects features of the individual reader's voice whilst speaking out loud, specifically, their regional accent.
